# Making waves: Why do we need ultra-permeable nanofiltration membranes for water treatment?

**DOI:** 10.1016/j.wroa.2023.100172

**Published:** 2023-02-18

**Authors:** Zhe Yang, Chenyue Wu, Chuyang Y. Tang

**Affiliations:** Department of Civil Engineering, the University of Hong Kong, Pokfulam, Hong Kong, SAR, PR China

**Keywords:** Ultra-permeable nanofiltration membranes, Water/wastewater treatment, New processes, Membrane materials

## Abstract

Over the last few decades, developing ultra-permeable nanofiltration (UPNF) membranes has been a focus research area to support NF-based water treatment. Nevertheless, there have been ongoing debates and doubts on the need for UPNF membranes. In this work, we share our perspectives on why UPNF membranes are desired for water treatment. We analyze the specific energy consumption (SEC) of NF processes under various application scenarios, which reveals the potential of UPNF membranes for reducing SEC by 1/3 to 2/3 depending on the prevailing transmembrane osmotic pressure difference. Furthermore, UPNF membranes could potentially enable new process opportunities. Vacuum-driven submerged NF-modules could be retrofitted to existing water/wastewater treatment plants, offering lower SEC and lower cost compared to conventional NF systems. Their use in submerged membrane bioreactors (NF-MBR) can recycle wastewater into high-quality permeate water, which enables energy-efficient water reuse in a single treatment step. The ability for retaining soluble organics may further extend the application of NF-MBR for anaerobic treatment of dilute municipal wastewater. Critical analysis of membrane development reveals huge rooms for UPNF membranes to attain improved selectivity and antifouling performance. Our perspective paper offers important insights for the future development of NF-based water treatment technology, which could potentially lead to a paradigm shift in this burgeoning field.

## Highlights

• Ultra-permeable nanofiltration (UPNF) is desired for water/wastewater treatment.
• UPNF offers major energy savings for many applications.
• UPNF enables new process designs for efficient water/wastewater treatment.
• UPNF membranes could potentially achieve high selectivity and low fouling.

## Graphical abstract

**Figure:**
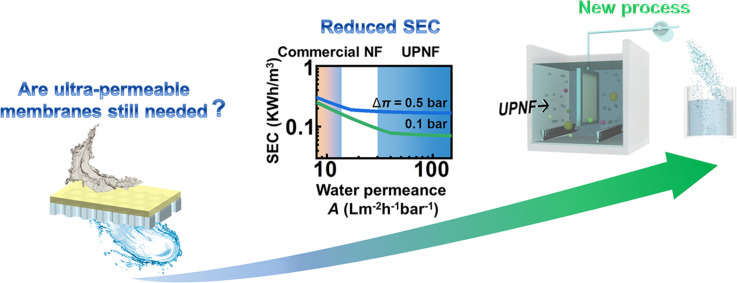

## 1 Introduction

Nanofiltration (NF) membranes, whose characteristics fall between “loose reverse osmosis (RO)” and “tight ultrafiltration (UF)” membranes (Wang et al., 2022), have been extensively employed in various water-related applications. Modern thin-film composite (TFC) polyamide NF membranes feature a fine pore size on the order of 1 nm, which allows them to selectively remove/transport low-molecular-weight compounds and ions via molecular sieving and/or charge interaction (Guo et al., 2021; Wang et al., 2022). The excellent ability to tune solute-water selectivity and solute-solute selectivity makes NF an attractive candidate for (1) the purification of surface water, groundwater, and brackish water (Lhassani et al., 2001; Su et al., 2021; Van der Bruggen et al., 2001), (2) the treatment or reuse of wastewater (Garcia-Ivars et al., 2017; Lan et al., 2018; Tang et al., 2018; Warsinger et al., 2018), and (3) the recovery or enrichment of valuable products (e.g., precious metals, nutrients, etc. (Ricci et al., 2015; Xie et al., 2016)).

**Fig. 1:**
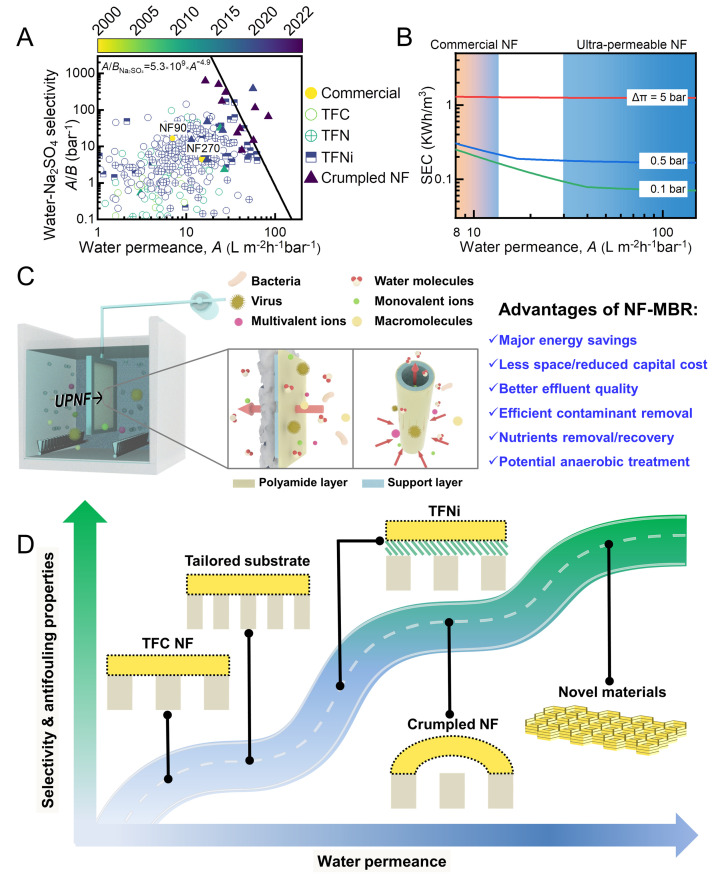
(A) The tradeoff between water permeance and water/Na_2_SO_4_ selectivity for NF membranes. The data for commercial NF membranes and lab-made TFC, thin-film nanocomposite (TFN), interlayered thin-film nanocomposite (TFNi), and crumpled NF membrane are mostly retrieved from Ref. (Yang et al., 2021), with some additional data obtained from Ref. (Teng et al., 2020). The data points for commercial NF membranes and lab-made NF membranes (including TFC, TFNi, and crumpled NF membranes) are color-mapped according to their publication year (spanning from 2000 to 2022). The solid line in the figure corresponds to the upper bound reported in Ref. (Yang et al., 2021).(B) Analysis of specific energy consumption (SEC) of a crossflow NF process under different transmembrane osmotic pressure differences (Δ*π* = 0.1, 0.5, and 5 bar). The analysis is performed in accordance with our previous works (Yang et al., 2021, 2020), and the following parameters are adopted: water flux = 40 Lm^−2^h^−1^; water recovery = 80%; pump efficiency = 80%. The analysis does not account for any energy recovery device (ERD) since ERD is rarely used for NF processes. (C) A schematic diagram illustrating the potential use of UPNF membranes (e.g., flat sheet type and hollow fiber type) in a submerged NF membrane bioreactor (NF-MBR). Their high water permeance allows UPNF membranes to be driven by a vacuum suction. Their ability to retain soluble organics could potentially extend the use of NF-MBR for anaerobic treatment of municipal wastewater. (D) The schematic diagram illustrating the advancement of water permeance, selectivity, and antifouling properties of UPNF membranes. Notable examples include TFNi membranes, UPNF using tailored (e.g., more porous) substrates, crumpled NF membranes, and some novel materials-based NF membranes.

NF technology has seen rapid advancements since the turn of the century. Many highly permeable and highly selective materials, such as aquaporins (Kumar et al., 2007), artificial water channels (Barboiu, 2012), and graphene-based materials (Nair et al., 2012), have been successfully incorporated into synthetic membranes (Lim et al., 2022). At the same time, new fabrication methods and novel membrane structures endow TFC polyamide membranes with greatly enhanced separation performance (Fig. 1A). In addition to the pursuit of high membrane selectivity, many researchers focus on the development of ultra-permeable NF (UPNF) membranes (Wang et al., 2022; Yang et al., 2021). Nevertheless, others are of the opinion that high water permeance plays a far less important role than selectivity (Shi et al., 2017; Werber et al., 2016a). Although this concern is legitimate for membrane processes involving high transmembrane osmotic pressure difference (Δ*π*) such as RO-based seawater desalination (Cohen-Tanugi et al., 2014; Elimelech and Phillip, 2011; Patel et al., 2020; Shi et al., 2017; Werber et al., 2016a, 2016b; Yang et al., 2021), many NF applications involves relatively low Δ*π* (Guo et al., 2021; Liu et al., 2022; Shao et al., 2022; Wang et al., 2022; Yang et al., 2021; Zhao et al., 2021), which leaves sufficient room for energy savings. Another common opinion is that, in view of the membrane permeance-selectivity tradeoff (Fig. 1A and (Geise et al., 2011; Park et al., 2017; Robeson, 1991; 2008; Werber et al., 2016b; Yang et al., 2019, 2021)), increasing membrane selectivity–rather than water permeance–is more important to enable process-favorable applications (Lu and Elimelech, 2021; Werber et al., 2016a).

The ongoing debates inspire us to critically analyze the existing literature and examine the untapped opportunities associated with UPNF. Herein, we provide our perspectives on why UPNF membranes are desired for water treatment. We show the potential energy savings offered by UPNF under various application scenarios. We further explore new process possibilities enabled by UPNF, which could potentially lead to a paradigm-shift in applying NF technology for water/wastewater treatment. Opportunities for novel membrane development are also highlighted. Our perspective paper offers critical insights for the future development of NF-based water treatment technology.

## 2 Why are UPNF membranes needed for water applications?

### 2.1 UPNF reduces specific energy consumption (SEC) for various applications

Researchers from different backgrounds often have different opinions on the value of high-permeance membranes. While some membrane makers believe that high water permanence can directly lead to major savings in SEC, a typical practitioner in seawater RO desalination would argue that only marginal reduction of SEC can be achieved. In reality, the available savings in SEC is strongly dependent on the transmembrane osmotic pressure difference Δ*π* (Fig. 1B). Unlike seawater RO desalination that deals with highly saline water (total dissolved solids TDS ∼= 32,000 ppm and Δ*π* ∼= 25 bar), many NF applications involve feedwaters with relatively low TDS and thus low osmotic pressure values (*π_feed_*). For example, a typical municipal wastewater may have a TDS of 500–1000 ppm, which translates into a *π_feed_* value of only 0.4–0.7 bar when treatment or reuse of municipal wastewater is considered. The applicable *π_feed_* value is even lower for typical surface water treatment and household tap water purification.

In Fig. 1B, we analyze the benefit of UPNF in terms of potential savings in SEC (Yang et al., 2020) using three different Δ*π* values: 0.1 bar (typical surface water scenario); 0.5 bar (representative of typical municipal wastewater and ground water); and 5 bar (relevant to brackish water and some saline industrial wastewater). Since the water permeance of commercially available NF membranes is generally in the range of 8 – 14 Lm^−2^h^−1^bar^−1^ (Fane et al., 2011), a value of 10 Lm^−2^h^−1^bar^−1^ is used for benchmarking purpose. Considering an UPNF membrane with a water permeance of 50 Lm^−2^h^−1^bar^−1^, a one-third reduction in SEC is achievable for Δ*π* = 0.5 bar; the available reduction is as much as 63.5% for Δ*π* = 0.1 bar. In contrast, the energy saving is only 2.7% for Δ*π* = 5 bar, largely due to the dominance of osmotic pressure over the transmembrane resistance.

It is worthwhile to note that, even for highly saline feedwaters with high *π_feed_* values, many applications may not require the removal of TDS. Instead, specific contaminants or valuable resources are targeted, such as the removal of fouling precursors in seawater pretreatment for desalination (Zhang et al., 2022) and the recovery of previous metals from metal plating wastewater (Ricci et al., 2015). For such cases, NF membranes can be designed to allow selective passage of TDS (e.g., with low retention of monovalent ions), which effectively reduces the transmembrane osmotic pressure difference toward greater energy savings. Our critical analysis reveals a huge potential of UPNF membranes for achieving major savings in SEC for a wide range of applications, which should be further explored in future developments.

### 2.2 UPNF enables new process opportunities

In addition to reduced SEC, UPNF membranes may enable new module/process opportunities. Constrained by their relatively low water permeance, existing commercial NF membranes are generally used in side-stream crossflow filtration systems with an applied hydraulic pressure typically in the range of 2 – 10 bar (Fane et al., 2011). High membrane water permeance could potentially allow the use of NF membranes in submerged filtration system driven by a partial vacuum (Fig. 1C). For example, using an UPNF membrane with a water permeance of 50–100 Lm^−2^h^−1^bar^−1^, the required vacuum suction could be as low as 0.1–0.2 bar for achieving a practically relevant water flux of 10 Lm^−2^h^−1^. Such a submerged UPNF membrane system offers multiple benefits. It allows easy retrofitting/upgrading of existing drinking water treatment plants and/or wastewater treatment plants. The elimination of pressure vessels and reduced land requirement could potentially save >10% in capital cost. Major energy savings may be achievable with the use of much lower transmembrane pressure. The possible use of hollow fiber UPNF membranes in submerged systems further enables greater membrane packing density and easier system operation.

An interesting potential application of UPNF membranes is membrane bioreactors for wastewater reclamation. Existing MBRs typically adopt porous microfiltration (MF) or UF membranes. Historically, the adoption of submerged MF/UF modules led to the wide-spread applications of MBRs for wastewater treatment (Meng et al., 2009, 2017; Tadkaew et al., 2011; Xiao et al., 2019; Yamamoto et al., 1989). However, due to the limited ability of MF/UF membranes for removing dissolved solutes such as nutrients (N and P), metals, and organic micropollutants, a polishing step (e.g., by RO) is often required for reuse purpose (Tay et al., 2018). One notable example is Singapore's Changi NEWater Plant, which recycles municipal wastewater into highly purified water through an MBR+RO treatment train (Tang et al., 2018). The adoption of UPNF membranes could enable the development of a submerged NF-MBR to achieve water reuse in a single treatment step, with UPNF membranes serving the dual roles of (1) separation of biomass and (2) removal of contaminants including dissolved ones (Fig. 1C). The high selectivity of NF membranes could greatly improve the effluent water quality. For organic micropollutants, the use of high-retention membranes allows their longer residence in the bioreactor, which may substantially improve their removal through the combined actions of membrane retention and biodegradation (Wang et al., 2018). Despite of these obvious benefits of NF-MBRs, nevertheless, membrane fouling could pose a critical challenge (Le-Clech et al., 2006). Therefore, future studies are needed to develop effective fouling control and cleaning strategies in NF-MBRs (Wang et al., 2014).

A further untapped opportunity is the development of anaerobic NF-MBRs for municipal wastewater treatment. Although it is difficult to apply conventional MBRs for the anaerobic treatment of municipal wastewaters with low organic strength, the use of high retention membranes could concentrate soluble organics in the bioreactor to make it an attractive option. Through the production of CH_4_ or H_2_, anaerobic NF-MBRs could potentially make wastewater treatment plants less energy intensive (or even converting them into net energy producers). In addition to energy recovery, the ability to retain N and P may also allow the use of NF-MBRs for nutrient recovery (Xie et al., 2016).

### 2.3 There is still huge room for developing UPNF membranes with desired selectivity and antifouling performance

Despite their vast untapped opportunities, UPNF membranes need to possess suitable selectivity and antifouling properties that are required for practical applications. Indeed, one prevailing argument is that membrane selectivity deserves priority over water permeance (Shi et al., 2017; Werber et al., 2016a). We are of the opinion that balanced combinations of selectivity and permeability are needed to suit different application requirements (Yang et al., 2021). Recent literature reports promising developments of high-performance polyamide NF membranes (Shao et al., 2022; Wang et al., 2022; Yang et al., 2020; Zuo et al., 2021). One notable strategy is NF membranes with crumpled surfaces (Fig. 1D), which can simultaneously increase the effective membrane filtration area and optimize the water transport pathways (Shao et al., 2022). As shown in Fig. 1A, some crumpled NF membranes surpass the previously reported upper bound with respect to water permeance and water/Na_2_SO_4_ selectivity. Interlayered thin-film nanocomposite (TFNi) membranes, featuring an interlayer sandwiched between the polyamide and substrate layers to improve the membrane formation and water transport pathways, also show promising combinations of permeance and selectivity (Dai et al., 2020; Yang et al., 2020). Recent studies also show that the use of more porous substrates (or interlayer-modified substrates) could enhance membrane water permeance and antifouling performance at the same time (Long et al., 2022; Wu et al., 2022). In the longer run, the development of novel/alternative desalination materials (Lim et al., 2022; Werber et al., 2016b) may provide greater rooms for simultaneously attainment of high water permeance, high selectivity, and excellent antifouling performance.

It is worthwhile to note that, although Fig. 1A only presents the upper bound for water/Na_2_SO_4_-based selectivity, practical applications may often mandate different water/solute selectivity and solute/solute selectivity based on the specific treatment goals. The future development of NF technology should specifically consider such application-oriented selectivity requirements. Taking water reuse as an example, the removal of harmful organic micropollutants is of critical importance to protect the public health. While this critical aspect is often neglected in traditional membrane development, some studies report the feasibility of simultaneous enhancement of pollutant removal and water permeance–revealing the huge room for future membrane development and optimization (Guo et al., 2022; Liu et al., 2022; Yang et al., 2020).

## 3 Conclusions

• The necessity of UPNF membranes has been long debated in the literature. We are of the opinion that UPNF membranes are desired for water and wastewater treatment.
• In terms of energy consumption, UPNF can offer an impressive SEC savings of 63.5% at a transmembrane osmotic pressure difference of 0.1 bar. Nevertheless, the available reduction in SEC becomes marginal at Δ*π* = 5 bar due to the dominance of osmotic pressure over the transmembrane resistance.
• UPNF could be potentially applied in submerged membrane bioreactors (NF-MBR) for wastewater treatment/reuse. Compared to side-stream crossflow configuration, submerged vacuum-driven UPNF features lower energy consumption and easier retrofitting/upgrading of water/wastewater treatment plants. The ability to retain soluble organics may further allow NF-MBR for the anaerobic treatment of municipal wastewater.
• Future development of UPNF membranes needs to simultaneously cater for water permeance, selectivity, and antifouling properties to meet the requirements for practical applications. Application specific requirements for selectivity along with permeance should be considered in membrane development and optimization.

## CRediT authorship contribution statement

**Zhe Yang:** Conceptualization, Formal analysis, Writing – original draft, Writing – review & editing. **Chenyue Wu:** Visualization. **Chuyang Y. Tang:** Conceptualization, Writing – original draft, Writing – review & editing.

## Declaration of Competing Interest

None.

## Data Availability

Data will be made available on request
